# Predictive value of non-high-density lipoprotein cholesterol to high-density lipoprotein cholesterol ratio for in-stent restenosis after drug-eluting stent implantation in patients with coronary heart disease

**DOI:** 10.1371/journal.pone.0356478

**Published:** 2026-08-31

**Authors:** Tilakezi Tuersun, Naibi Mijiti, Guzailinuer Wubulikasimu, Ali Maimaitiming

**Affiliations:** 1 Department of Cardiology, The First People’s Hospital of Kashi Prefecture, Kashi, China; 2 Clinical Laboratory Center, The First People’s Hospital of Kashi Prefecture, Kashi, China; IPSurface Canada Inc, CANADA

## Abstract

**Background & Objective:**

In-stent restenosis (ISR) remains a major obstacle after drug-eluting stent (DES) implantation in patients with coronary heart disease (CHD), in which dyslipidemia play important roles. The non-high-density lipoprotein cholesterol to high-density lipoprotein cholesterol ratio (NHHR) have emerged as potential markers of both lipid metabolism and atherosclerosis. However, the association between NHHR and ISR after DES implantation in patients with CHD remains unclear. Consequently, the objective of this study was to determine the association between NHHR and ISR and its predictive value among CHD patients after DES implantation.

**Methods:**

The clinical data of 496 patients with CHD who underwent DES implantation in the Department of Cardiology of the First People ‘s Hospital of Kashgar from January 2019 to December 2024 were collected. All patients completed postoperative follow-up, with a median follow-up time of 6 months. Patients were divided into three groups based on NHHR tertiles: Q1 (25th percentile), Q2 (50th percentile), and Q3 (75th percentile). Baseline characteristics were compared among groups. Restricted cubic spline (RCS) plots were used to explore the association between NHHR and ISR. Binary logistic regression analysis was performed to identify independent risk factors for ISR. Finally, receiver operating characteristic (ROC) curves were generated to evaluate the predictive performance of NHHR for ISR.

**Results:**

There were significant differences in age, total cholesterol (TC), high-density lipoprotein cholesterol (HDL-C), low-density lipoprotein cholesterol (LDL-C), stent length, number of stents and proportion of right coronary stent implantation in different NHHR groups (P < 0.05). RCS and logistic regression analyses revealed that NHHR and TC were independent risk factors for ISR (OR>1, P < 0.05). ROC curve analysis demonstrated that NHHR had superior predictive ability for ISR (AUC 0.702, 95% CI 0.630–0.775) compared to TC (AUC 0.639, 95% CI 0.564–0.714) and HDL-C (AUC 0.567, 95% CI 0.490–0.643).

**Conclusion:**

NHHR and TC are independent risk factors for ISR after DES implantation in CHD patients. Compared to traditional lipid markers TC and HDL-C, NHHR exhibits superior predictive value for ISR and may serve as a key clinical indicator for risk stratification.

## 1. Background

Coronary heart disease (CHD) is a leading cause of global morbidity and mortality and remains the primary cause of death in China [1]. As a guideline-recommended essential treatment for CHD, percutaneous coronary intervention (PCI) has been widely adopted in clinical practice [2]. With the dramatic increase in PCI procedures, the incidence of in-stent restenosis (ISR) post-PCI has shown an upward trend. ISR represents one of the common serious complications following PCI. Although drug-eluting stents demonstrate significantly improved efficacy and safety compared to traditional bare-metal stents, approximately 5%−10% of patients still develop ISR in real-world clinical practice [3–5]. Once ISR occurs, it may lead to serious cardiovascular events and increased risk of repeat revascularization [6,7]. Therefore, screening for biological markers with high sensitivity to achieve early diagnosis and implementing timely interventions are crucial for improving the prognosis of ISR patients.

Recent studies increasingly demonstrate that compared to traditional low-density lipoprotein cholesterol (LDL-C) and high-density lipoprotein cholesterol (HDL-C), the non-high-density lipoprotein cholesterol to high-density lipoprotein cholesterol ratio (NHHR) better reflects dyslipidemia and shows a stronger correlation with cardiovascular disease risk [8,9]. However, no studies to date have reported its role in ISR. Therefore, this study aims to investigate the relationship between NHHR and ISR after drug-eluting stent implantation for coronary heart disease, and to evaluate its predictive value for ISR.

## 2. Methods

### 2.1. Study population

This single-center, retrospective study enrolled 496 consecutive patients with CHD who underwent drug-eluting stent implantation at the Department of Cardiology, the First People’s Hospital of Kashgar, between January 2019 and December 2024. All patients completed a standardized postoperative follow-up with a median duration of 6 months. The cohort comprised 386 males (77.8%) and 110 females (22.2%), with a mean age of 62.45 ± 10.28 years. The medical records of eligible participants were retrieved from the hospital medical record department on 25 December 2024. The authors had access to identifiable personal information (e.g., name, ID number) during data retrieval, and all personal identifiers were removed immediately after data extraction to ensure participant confidentiality. The study followed the ethical guidelines of the Declaration of Helsinki and received approval from the Ethics Committee of the First People’s Hospital of Kashgar Prefecture ([2023] quick review research No.02, approved on June 23, 2023).

The inclusion criteria were: 1) age between 18 and 80 years; 2) completion of follow-up coronary angiography at 6 months after DES implantation; and 3) provision of written informed consent. Key exclusion criteria included: 1) incomplete medical records; 2) chronic total occlusion lesions in the stented vessels; and 3) a history of severe hepatic or renal insufficiency, malignant tumors, or rheumatic immune diseases. All DES implanted in this cohort were second-generation, non-degradable polymer-based platforms (predominantly everolimus-, zotarolimus-, and sirolimus-eluting stents); biodegradable-polymer DES are not yet routinely available in our region, and accordingly the present analysis pertains exclusively to non-degradable polymer DES.

The NHHR was calculated as follows: NHHR = (Total Cholesterol – HDL-C)/ HDL-C.

ISR was defined as ≥50% luminal diameter narrowing within the stent segment or its proximal and distal 5 mm margins through follow-up coronary angiography. ISR events were strictly distinguished from acute, subacute, and late stent thrombosis using the Academic Research Consortium (ARC) consensus definitions [10]; thrombotic occlusions presenting as abrupt luminal closure with angiographic thrombus burden, typically accompanied by acute ischaemic symptoms or biomarker elevation, were not classified as ISR. Among the cohort, 91 patients (18.3%) underwent intravascular ultrasound (IVUS) or optical coherence tomography (OCT) at the time of repeat angiography to characterise the mechanism of restenosis and to differentiate neointimal hyperplasia from neoatherosclerosis or residual thrombus, and fractional flow reserve (FFR) was obtained in 24 patients (4.8%) with intermediate-grade angiographic restenosis (40–70%) to confirm haemodynamic significance, in line with current expert consensus on intracoronary imaging and physiology [11]. Restenotic lesions were further categorised angiographically using the Mehran classification [12] into focal (Pattern I, lesion length ≤10 mm) and diffuse (Patterns II–IV, lesion length >10 mm) ISR for subgroup analysis.

### 2.2. Data collection

Clinical data were systematically extracted from the hospital’s electronic medical records. Collected variables included demographic characteristics (sex, age), cardiovascular risk factors (smoking history, alcohol consumption, hypertension, diabetes mellitus), and laboratory parameters (HDL-C, low-density lipoprotein cholesterol [LDL-C], total cholesterol [TC], triglycerides, serum creatinine, and blood glucose). Procedural details, such as the total stent length, number of stents implanted, and the specific coronary arteries with stenotic lesions, were recorded. Medication use at discharge was also documented, including enteric-coated aspirin, clopidogrel, ticagrelor, dual antiplatelet therapy (DAPT), statins, angiotensin-converting enzyme inhibitors/angiotensin II receptor blockers (ACEI/ARB), and β-blockers. Additional procedural and anatomical covariates recorded included reference vessel diameter, with small-vessel disease pre-specified as a reference diameter <2.75 mm and large-vessel disease as ≥2.75 mm at the target lesion site, lesion length, the use of overlapping stents (defined as ≥2 stents implanted in the same vessel with ≥1 mm overlap of stent struts), and the angiographic ISR pattern (focal versus diffuse) for those who developed restenosis. Lipid-lowering co-therapies beyond statins were also documented, encompassing ezetimibe, proprotein convertase subtilisin/kexin type 9 inhibitors (PCSK9i, evolocumab and alirocumab), and bempedoic acid, in line with contemporary lipid management guidelines [13]. Clinical outcomes assessed at follow-up included symptomatic status at the time of repeat angiography (typical anginal chest pain, dyspnoea on exertion, or anginal-equivalent symptoms versus asymptomatic surveillance angiography), target-lesion revascularisation (TLR), target-vessel revascularisation (TVR), and major adverse cardiovascular events (MACE; composite of cardiovascular death, non-fatal myocardial infarction, and TLR).

### 2.3. Statistical analysis

Continuous variables are presented as mean ± standard deviation if normally distributed, or as median [interquartile range] otherwise. Group comparisons for normally distributed data were performed using the Student’s t-test (two groups) or one-way ANOVA (multiple groups); the Mann-Whitney U or Kruskal-Wallis test was used for non-normally distributed data. Categorical variables are expressed as numbers (percentages) and were compared using the Chi-square test or Fisher’s exact test, as appropriate. The relationship between NHHR and ISR was initially visualized and analyzed using restricted cubic splines (RCS) with the rms package in R. Subsequently, univariate and multivariate logistic regression models were constructed to quantify the association between NHHR levels and ISR risk. The predictive performance of NHHR for ISR was evaluated using receiver operating characteristic (ROC) curve analysis, and the area under the curve (AUC) was calculated. All analyses were conducted using R software (version 4.4.1), and a two-sided P-value < 0.05 was considered statistically significant. Pre-specified subgroup analyses were performed by reference vessel size (small vs. large), angiographic ISR pattern (focal vs. diffuse), and overlapping versus non-overlapping stent placement, with effect modification assessed using likelihood-ratio tests for interaction. To compare the incremental predictive value of NHHR over individual lipid parameters (LDL-C, HDL-C, TC, and the LDL-C/HDL-C ratio), parallel multivariable models were constructed and continuous net reclassification improvement (NRI) and integrated discrimination improvement (IDI) were calculated, with model fit additionally compared using the Akaike information criterion (AIC). Calibration of the final NHHR-based model was assessed by the Hosmer–Lemeshow goodness-of-fit test. The required sample size was estimated a priori using the rule of at least ten outcome events per included covariate for the multivariable logistic model and confirmed by simulation, indicating that the achieved cohort of 496 patients with 56 ISR events provided approximately 85% statistical power to detect an odds ratio of 1.40 per unit increase in NHHR at a two-sided α of 0.05.

## 3. Results

### 3.1. Baseline characteristics stratified by NHHR tertiles

Patients were categorized into tertiles based on their NHHR values: Q1 (1.16–2.85, n = 165), Q2 (2.85–3.91, n = 165), and Q3 (3.91–9.00, n = 166). The mean age significantly decreased across the increasing NHHR tertiles (P = 0.008). As expected, significant differences were observed in lipid profiles, with stepwise increases in TC and LDL-C and a decrease in HDL-C from Q1 to Q3 (all P < 0.001). Procedural complexity, indicated by total stent length and the number of stents implanted, was also higher in the upper NHHR tertile (both P < 0.001). Furthermore, the proportion of patients with a stent implanted in the right coronary artery differed significantly among the groups (P < 0.001). The baseline characteristics are detailed in Table 1. With respect to anatomical and procedural complexity, 168 patients (33.9%) had small-vessel target lesions (reference diameter <2.75 mm) and 89 patients (17.9%) received overlapping stents; the proportion of small-vessel disease did not differ significantly across NHHR tertiles (32.7% vs. 33.3% vs. 35.5% in Q1, Q2 and Q3, respectively; P = 0.851), whereas overlapping-stent use was modestly more frequent in the upper tertile (13.9% vs. 16.4% vs. 23.5%; P = 0.046). Among the 56 patients who developed ISR, the angiographic restenosis pattern comprised 31 focal (55.4%) and 25 diffuse (44.6%) lesions. The mean LDL-C/HDL-C ratio across the cohort was 2.93 ± 1.13 and increased stepwise from Q1 to Q3 (1.99 ± 0.62, 2.81 ± 0.78, and 3.97 ± 1.25, respectively; P < 0.001). Use of non-statin lipid-lowering therapy at discharge was modest, including ezetimibe in 47 patients (9.5%), PCSK9 inhibitors in 11 patients (2.2%), and bempedoic acid in 3 patients (0.6%); the proportion of patients receiving any non-statin add-on therapy did not differ significantly across NHHR tertiles (10.3% vs. 11.5% vs. 14.5% in Q1, Q2 and Q3, respectively; P = 0.453). At repeat angiography, 38 of the 56 ISR patients (67.9%) presented with anginal symptoms, whereas 18 (32.1%) were detected during routine protocol-driven follow-up despite being clinically asymptomatic.

**Table 1:** Baseline Clinical Characteristics of the Study Population Stratified by NHHR Tertiles.

Factor	Overall Population (n = 496)	Q1 group [1.16, 2.85] (n = 165)	Q2 group [2.85, 3.91] (n = 165)	Q3 group [3.91,9.00] (n = 166)	F/χ² value	P value
Age (years)	62.45 ± 10.28	64.25 ± 10.93	62.36 ± 9.28	60.74 ± 10.30	4.924	0.008
Gender					1.791	0.408
Female	110 (22.2%)	39 (23.6%)	40 (24.2%)	31 (18.7%)		
Male	386 (77.8%)	126 (76.4%)	125 (75.8%)	135 (81.3%)		
Smoking history, n (%)	228 (46.0%)	76 (46.1%)	66 (40.0%)	86 (51.8%)	4.646	0.098
Alcohol consumption history, n (%)	101 (20.4%)	39 (23.6%)	28 (17.0%)	34 (20.5%)	2.263	0.323
History of hypertension, n (%)	321 (64.7%)	112 (67.9%)	101 (61.2%)	108 (65.1%)	1.619	0.445
History of diabetes mellitus, n (%)	153 (30.8%)	48 (29.1%)	46 (27.9%)	59 (35.5%)	2.636	0.268
TC, mmol/l	4.40 ± 1.18	3.66 ± 0.86	4.24 ± 0.87	5.29 ± 1.16	118.616	< 0.001
HDL-C, mmol/l	1.01 ± 0.23	1.12 ± 0.26	0.98 ± 0.20	0.92 ± 0.18	39.335	< 0.001
LDL-C, mmol/l	2.80 ± 0.89	2.17 ± 0.58	2.70 ± 0.58	3.53 ± 0.86	165.213	< 0.001
Serum creatinine, μmol/L	91.56 ± 70.96	83.91 ± 23.06	99.26 ± 101.69	91.51 ± 64.67	1.938	0.145
Stent length, mm	44.74 ± 32.01	36.14 ± 25.49	45.02 ± 32.82	53.01 ± 34.86	12.008	< 0.001
Number of stents implanted	2.03 ± 1.24	1.75 ± 1.01	2.01 ± 1.27	2.34 ± 1.33	10.106	< 0.001
Stenotic vessel						
Left main coronary artery, n (%)	38 (7.7%)	13 (7.9%)	15 (9.1%)	10 (6.0%)	1.117	0.572
Left anterior descending artery, n (%)	323 (65.1%)	113 (68.5%)	106 (64.2%)	104 (62.7%)	1.324	0.516
Circumflex artery, n (%)	118 (23.8%)	33 (20.0%)	40 (24.2%)	45 (27.1%)	2.334	0.311
Right coronary artery, n (%)	208 (41.9%)	50 (30.3%)	81 (49.1%)	77 (46.4%)	13.989	< 0.001
Medication status						
Enteric-coated aspirin tablets, n (%)	472 (95.2%)	152 (92.1%)	161 (97.6%)	159 (95.8%)	5.540	0.063
Clopidogrel, n (%)	408 (82.3%)	141 (85.5%)	132 (80.0%)	135 (81.3%)	1.831	0.400
Ticagrelor, n (%)	99 (20.0%)	23 (13.9%)	39 (23.6%)	37 (22.3%)	5.703	0.058
DAPT, n (%)	477 (96.2%)	155 (93.9%)	161 (97.6%)	161 (97.0%)	3.415	0.181
Statins, n (%)	482 (97.2%)	159 (96.4%)	159 (96.4%)	164 (98.8%)	2.785	0.295
ACEI/ARB, n (%)	395 (79.6%)	130 (78.8%)	125 (75.8%)	140 (84.3%)	3.866	0.145
β-blockers, n (%)	438 (88.3%)	138 (83.6%)	147 (89.1%)	153 (92.2%)	5.981	0.050
Medication adherence, n (%)	228 (45.96%)	81 (49.09%)	78 (47.27%)	69 (41.57%)	2.056	0.561

Note: TC, total cholesterol; HDL-C, high-density lipoprotein cholesterol; LDL-C, low-density lipoprotein cholesterol; DAPT: dual antiplatelet therapy; ACEI/ARB: angiotensin-converting enzyme inhibitors/angiotensin II receptor blockers.

### 3.2. Association of NHHR, HDL-C, and TC with ISR

**Fig 1:**
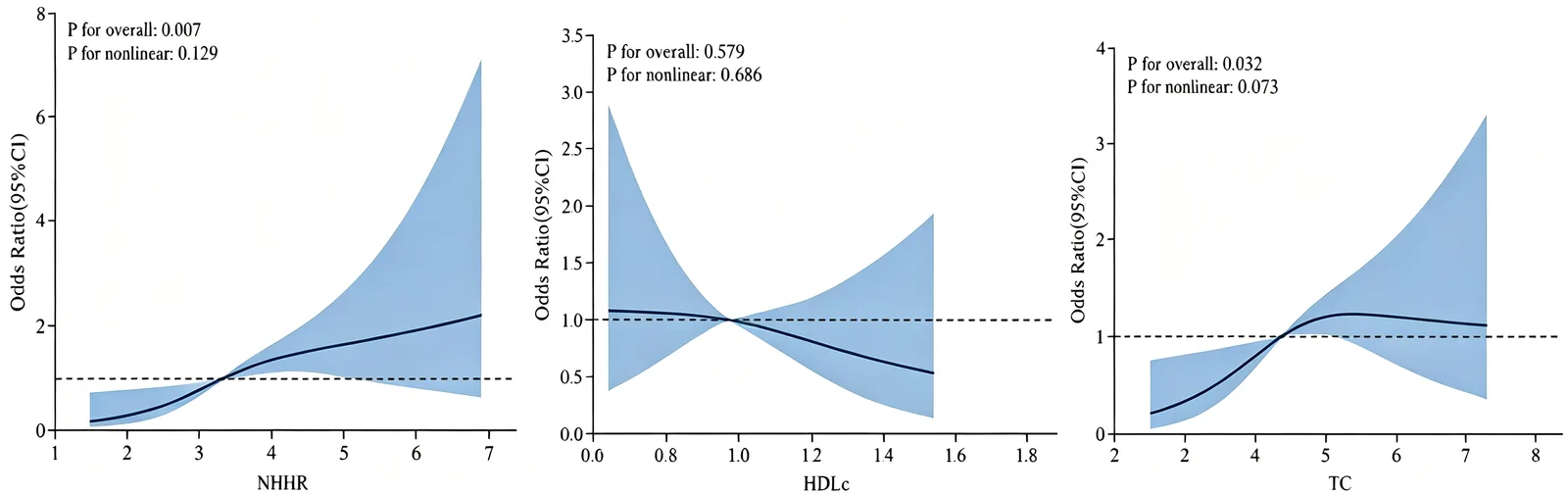
RCS curve analysis of the association between NHHR, HDL-C, and TC with ISR.

Restricted cubic spline (RCS) curves were plotted to analyze the associations of NHHR, HDL-C, and TC with ISR. These analyses revealed linear relationships for all three indicators with ISR (P for nonlinearity>0.05). NHHR and TC levels exhibited a significant positive correlation with ISR (overall P < 0.05). Although HDL-C also demonstrated a linear relationship with ISR, the association was not statistically significant (overall P = 0.579), as detailed in Fig 1.

### 3.3. Risk factors for ISR

In univariate analysis (Model 1), both NHHR (OR 1.39, 95% CI 1.14–1.71) and TC (OR 1.32, 95% CI 1.06–1.64) were significant predictors of ISR. These associations remained robust after sequential adjustment for age and sex (Model 2), and further comprehensive adjustment for smoking history, alcohol consumption, hypertension, diabetes, medication use (including aspirin, clopidogrel, ticagrelor, statins, dual antiplatelet therapy, ACEI/ARB, and beta-blockers), total stent length, number of implanted stents, and stenotic vessels (left main, left anterior descending, circumflex, and right coronary arteries) in Model 3. In the fully adjusted model (Model 3), every unit increase in NHHR was associated with a 47% higher risk of ISR (OR 1.47, 95% CI 1.15–1.87). When analyzed by tertiles, patients in the highest NHHR tertile (Q3) had a nearly fourfold increased risk of ISR (OR 3.91, 95% CI 1.58–9.69) compared to those in the lowest tertile (Q1). HDL-C was not an independent risk factor in any model. The results of the logistic regression analyses are presented in Table 2. In a parallel multivariable model substituting NHHR with the LDL-C/HDL-C ratio, the latter was also independently associated with ISR in Model 3 (OR 1.34 per unit, 95% CI 1.07–1.68; P = 0.012); however, both the magnitude of association and goodness of fit were modestly inferior to those of NHHR (Akaike information criterion 358.7 for the LDL-C/HDL-C model vs. 351.4 for the NHHR model). LDL-C analysed alone yielded only a borderline association after full adjustment (OR 1.27 per mmol/L, 95% CI 0.99–1.62; P = 0.058), supporting the integrative advantage of NHHR over LDL-C considered in isolation.

**Table 2:** Analysis of risk factors for ISR after drug-eluting stent implantation for CHD.

Variable	Proportion of ISR occurrence	Model 1		Model 2		Model 3	
		OR(95%CI)	P value	OR(95%CI)	P value	OR(95%CI)	P value
NHHR	56/496	1.39 (1.14, 1.71)	0.001	1.49 (1.20, 1.84)	<0.001	1.47 (1.15, 1.87)	0.002
TC, mmol/l	56/496	1.32 (1.06, 1.64)	0.013	1.30 (1.04, 1.62)	0.023	1.31 (1.03, 1.66)	0.031
HDL-C, mmol/l	56/496	0.56 (0.16, 1.95)	0.361	0.31 (0.08, 1.19)	0.088	0.47 (0.11, 2.06)	0.320
Group							
Q1	8/165	Ref.		Ref.		Ref.	
Q2	21/165	2.86 (1.23, 6.66)	0.015	3.03 (1.29, 7.11)	0.011	2.98 (1.18, 7.48)	0.020
Q3	27/166	3.81 (1.68, 8.67)	0.001	4.34 (1.88, 9.99)	<0.001	3.91 (1.58, 9.69)	0.003

Note: Model 1: Unadjusted for any confounding factors; Model 2: Adjusted for gender and age; Model 3: Model 2 with additional adjustments for smoking history, history of alcohol consumption, history of hypertension, history of diabetes mellitus, medication status (enteric-coated aspirin tablets, clopidogrel, ticagrelor, statins, DAPT, ACEI/ARB, and β-blockers), total stent implantation length, number of stents implanted, and stenotic vessel (left main coronary artery, left anterior descending artery, circumflex artery, and right coronary artery). TC, total cholesterol; HDL-C: high-density lipoprotein cholesterol.

### 3.4. Predictive value of NHHR for ISR

**Fig 2:**
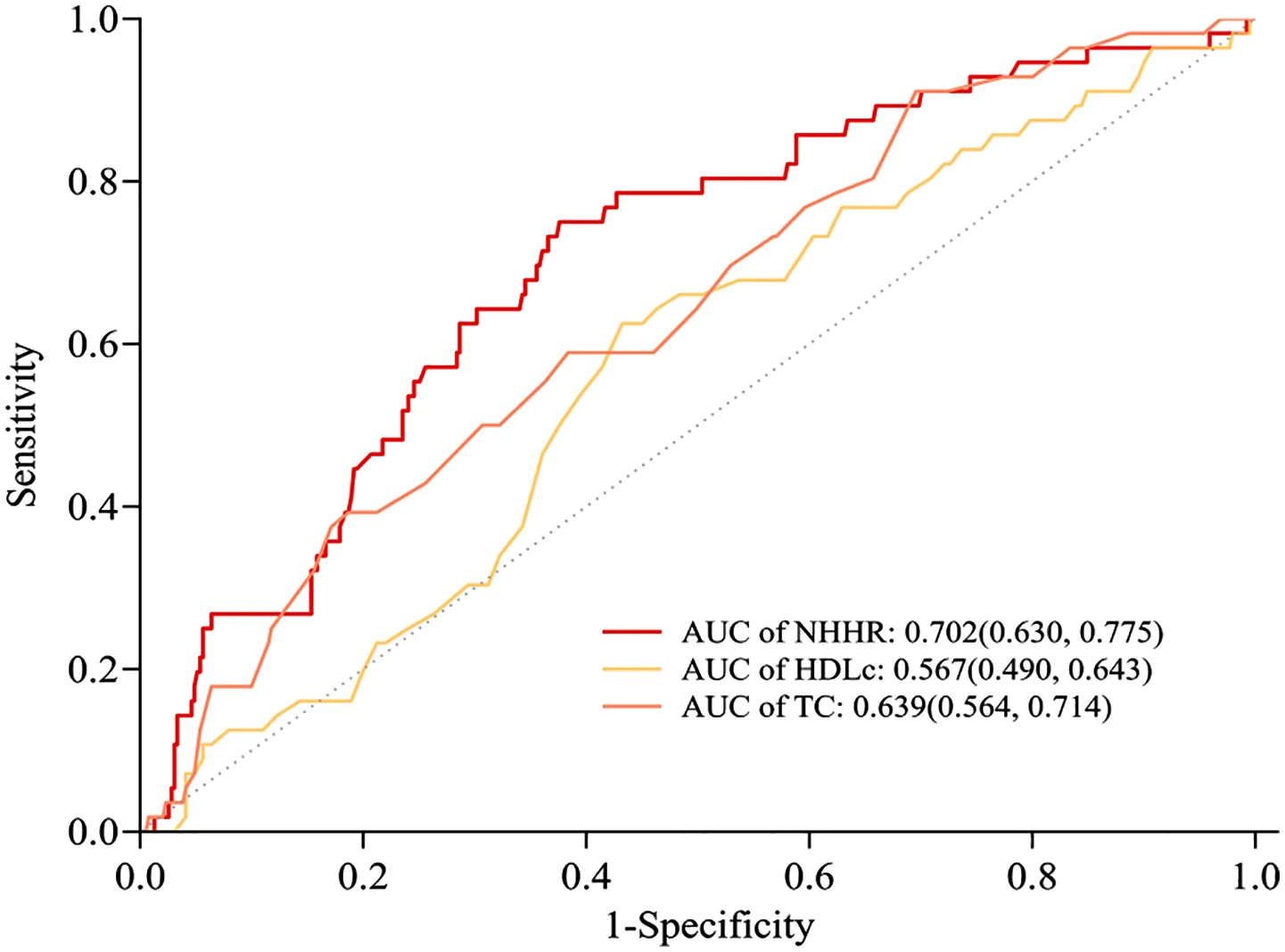
ROC curve analysis of the predictive value of NHHR, HDL-C, and TC for ISR.

ROC curve analysis demonstrated that NHHR had superior predictive ability for ISR (AUC 0.702, 95% CI 0.630–0.775) compared to TC (AUC 0.639, 95% CI 0.564–0.714) and HDL-C (AUC 0.567, 95% CI 0.490–0.643). The optimal NHHR cutoff value for predicting ISR was 3.383, which provided a sensitivity of 75.0% and a specificity of 62.4% (Table 3, Fig 2). When the predictive performance of NHHR was directly compared with that of the LDL-C/HDL-C ratio (AUC 0.671, 95% CI 0.594–0.748) and LDL-C alone (AUC 0.622, 95% CI 0.546–0.698), NHHR demonstrated superior discrimination, with a continuous net reclassification improvement (NRI) of 0.218 (95% CI 0.054–0.382; P = 0.009) and an integrated discrimination improvement (IDI) of 0.024 (95% CI 0.006–0.042; P = 0.008) over the LDL-C/HDL-C model. The Hosmer–Lemeshow goodness-of-fit test indicated good calibration of the NHHR-based model (χ² = 6.12, P = 0.634).

**Table 3:** Predictive value of NHHR, HDL-C, and TC for ISR.

Variable	AUC(95%CI)	Sensitivity	Specificity	Youden index	Optimal cutoff value
NHHR	0.702(0.630, 0.775)	0.750	0.624	0.374	3.383
HDL-C	0.567(0.490, 0.643)	0.568	0.625	0.193	0.965
TC	0.639(0.564, 0.714)	0.911	0.304	0.215	3.550

### 3.5. Subgroup analyses and clinical outcomes

Pre-specified subgroup analyses confirmed the consistency of the NHHR–ISR association across clinically relevant strata. The fully adjusted odds ratio for ISR per unit increase in NHHR was 1.51 (95% CI 1.10–2.07) in patients with small-vessel disease and 1.45 (95% CI 1.08–1.95) in those with large-vessel disease (P for interaction = 0.812). Stratification by ISR pattern showed that elevated NHHR was associated with both focal (OR 1.42, 95% CI 1.06–1.90) and diffuse (OR 1.55, 95% CI 1.11–2.16) restenosis, with diffuse ISR exhibiting a numerically stronger association though without statistically significant heterogeneity (P for interaction = 0.387). Among the 89 patients receiving overlapping stents, the ISR rate was 19.1% (17/89) compared with 9.6% (39/407) in those receiving non-overlapping stents (P = 0.010); within this overlapping-stent subgroup, the association between NHHR and ISR remained robust (OR 1.62, 95% CI 1.04–2.52). With regard to clinical outcomes during follow-up, TLR was performed in 31 patients (6.3%), TVR in 38 patients (7.7%), and the composite MACE endpoint occurred in 44 patients (8.9%); patients in the highest NHHR tertile experienced a significantly higher rate of MACE compared with the lowest tertile (13.9% vs. 4.2%; log-rank P = 0.002), reinforcing the clinical relevance of the NHHR-defined risk gradient. Among patients who developed ISR, 67.9% (38/56) presented with anginal symptoms at follow-up while 32.1% (18/56) were detected on protocol-driven surveillance angiography despite being asymptomatic, and the magnitude of NHHR elevation did not differ significantly between symptomatic and asymptomatic ISR (mean NHHR 4.21 ± 1.31 vs. 4.08 ± 1.24; P = 0.731), suggesting that the NHHR–ISR association is independent of clinical presentation.

## 4. Discussion

CHD remains a leading cause of global morbidity and mortality. While dyslipidemia—specifically, elevated LDL-C—is a well-established risk factor, a substantial residual cardiovascular risk persists even after achieving guideline-recommended LDL-C targets [14,15]. This underscores the critical role of other lipid parameters in the pathogenesis of CHD. Among emerging biomarkers, NHHR has recently gained prominence. Evidence suggests that NHHR may be superior to either non-HDL-C or LDL-C alone in predicting cardiovascular risk in high-risk populations, including those with diabetes mellitus, end-stage renal disease, and metabolic syndrome [16–18]. Pathophysiologically, non-HDL-C represents the total burden of atherogenic lipoproteins, including LDL-C, very-low-density lipoprotein cholesterol, intermediate-density lipoprotein, lipoprotein(a), and chylomicron remnants. Thus, the NHHR, by integrating atherogenic and atheroprotective components, provides a more holistic reflection of the overall atherogenic risk profile.

To our knowledge, this is the first study to identify NHHR as an independent risk factor for ISR following drug-eluting stent implantation in patients with CHD. This finding offers a novel perspective for stratifying ISR risk. Our results are consistent with a growing body of evidence underscoring the significant predictive value of NHHR for cardiovascular and metabolic disorders [18,19]. For instance, an analysis of the National Health and Nutrition Examination Survey (NHANES) data (2001–2018) established a significant positive correlation between NHHR and hypertension [20]. In particular, our results closely parallel those reported by Chen et al. [21] in a contemporaneous Chinese cohort undergoing PCI, in which higher NHHR was an independent predictor of ISR with a comparable effect size, lending external validity to NHHR as a lipid-based marker of post-PCI restenosis risk across distinct populations. Furthermore, NHHR has been positively associated with the risk of acute coronary syndrome [22]. Work by Ya Li et al. [23] suggests a negative correlation between NHHR and the development of coronary collateral circulation, implying a potential role for NHHR in coronary microvascular dysfunction. Similarly, a study by Liu M et al. [24] confirmed that NHHR effectively predicts the risk of adverse cardiovascular events and all-cause mortality in patients with type 2 diabetes mellitus, with higher values indicating greater risk. Beyond cardiovascular outcomes, NHHR also serves as an independent risk factor for diabetic nephropathy, with each unit increase associated with a 6% rise in disease prevalence [25]. The consistency between these diverse findings and the results of our present study further validates the pivotal role of NHHR across the spectrum of cardiovascular diseases and their complications.

Furthermore, this study established the superior predictive value of the non-high-density lipoprotein cholesterol to HDL-C ratio (NHHR) for in-stent restenosis (ISR) following drug-eluting stent implantation in CHD patients, compared to traditional lipid parameters such as total cholesterol (TC) and HDL-C. The underlying rationale for this superiority is multifactorial. First, NHHR provides a more holistic assessment of the atherogenic–antiatherogenic lipid balance. Unlike individual parameters, NHHR incorporates both the atherogenic burden, represented by non-HDL-C (which encompasses all pro-atherogenic lipoproteins), and the protective capacity of HDL-C, which mediates cardiovascular benefits through reverse cholesterol transport, anti-inflammatory, and antioxidant effects [26,27]. By integrating these opposing forces into a single ratio, NHHR more accurately reflects the net dynamic equilibrium central to the atherosclerotic process, a key pathological precursor to ISR. Second, the pathophysiological pathways implicated in ISR are directly influenced by the components of NHHR. Elevated non-HDL-C levels are closely linked to enhanced inflammatory responses and oxidative stress [28,29], which are pivotal mechanisms in ISR pathogenesis. Subsequent to stent-induced vascular injury, these processes drive the proliferation of vascular smooth muscle cells and neointimal hyperplasia, the fundamental processes leading to ISR [30]. Third, NHHR is implicated in plaque destabilization, a critical determinant of clinical events. The presence of unstable coronary plaques is a strong predictor of ISR development [31]. Notably, elevated NHHR has been associated with an increased prevalence of unstable plaque features in elderly patients with coronary artery disease [32], suggesting a mechanism by which NHHR may promote ISR through compromising plaque stability. Finally, NHHR serves as a sensitive integrator of systemic metabolic risk. It is strongly correlated with various metabolic abnormalities, including metabolic syndrome, thyroid dysfunction, and diabetes mellitus [33–35]. These conditions are established risk factors for both CHD and ISR, exacerbating risk through mechanisms such as endothelial dysfunction and pro-thrombotic states. Traditional lipid parameters like TC and HDL-C are less sensitive to these underlying metabolic disturbances, whereas NHHR effectively captures this integrated risk profile. In summary, by concurrently reflecting atherogenic burden, anti-atherogenic protection, key ISR-pathogenic mechanisms, and overall metabolic dysregulation, NHHR demonstrates a clear predictive advantage over conventional lipid metrics for ISR in this patient population.

This study has several limitations that should be considered. First, its single-center, retrospective design inherently carries potential risks of selection and information bias. Second, the study cohort was recruited from a single center in the Kashgar region, featuring a moderate sample size and a population with specific geographical and ethnic characteristics; consequently, the generalizability of our findings requires validation in larger, multi-center, prospective studies. Third, the assessment of medication adherence relied on medical record reviews and patient follow-up inquiries, which is subject to a degree of subjectivity. Fourth, the median follow-up duration was 6 months, capturing mid-term ISR risk but precluding an evaluation of NHHR for predicting very late ISR (e.g., beyond one year). Finally, although our multivariate models adjusted for a wide range of known confounders, the potential influence of unmeasured or residual confounding factors, such as specific inflammatory markers or genetic polymorphisms, cannot be entirely ruled out. Therefore, future studies with more rigorous designs, larger sample sizes, and comprehensive biomarker profiling are warranted to confirm the causal relationship between NHHR and ISR and to establish its definitive predictive utility. In addition, although the angiographic definition of ISR remains the established standard, the absence of routine intracoronary imaging and physiology assessment in the majority of patients precludes a granular characterisation of the underlying restenosis mechanisms (such as neointimal hyperplasia versus neoatherosclerosis) and may have under-detected functionally significant restenosis in patients with intermediate-grade angiographic narrowing. Furthermore, as biodegradable-polymer DES were not available in our centre during the study period, the present findings should be interpreted as applying specifically to non-degradable polymer DES, and further studies are required to determine whether the NHHR–ISR relationship extends to biodegradable-polymer platforms.

In conclusion, the NHHR is not only an independent risk factor for ISR after drug-eluting stent implantation in CHD patients, but also demonstrates superior efficacy to traditional lipid parameters such as HDL-C and TC in predicting ISR. This finding not only provides a more effective indicator for clinical prediction of ISR risk, but also offers a novel direction for further exploration into the pathogenesis of ISR and the development of intervention strategies.

## References

[1] Ren Y, Yang H, Browning C, Thomas S, Liu M. Prevalence of depression in coronary heart disease in China: a systematic review and meta-analysis. Chin Med J (Engl). 2014;127(16):2991–8. doi: 10.3760/cma.j.issn.0366-6999.20140036 25131240

[2] Rao SV, O’Donoghue ML, Ruel M, Rab T, Tamis-Holland JE, Alexander JH, et al. 2025 ACC/AHA/ACEP/NAEMSP/SCAI Guideline for the Management of Patients With Acute Coronary Syndromes: A Report of the American College of Cardiology/American Heart Association Joint Committee on Clinical Practice Guidelines. J Am Coll Cardiol. 2025;85(22):2135–237. doi: 10.1016/j.jacc.2024.11.009 40013746

[3] Giustino G, Colombo A, Camaj A, Yasumura K, Mehran R, Stone GW, et al. Coronary in-stent restenosis: JACC state-of-the-art review. J Am Coll Cardiol. 2022;80(4):348–72. doi: 10.1016/j.jacc.2022.05.017 35863852

[4] Pelliccia F, Zimarino M, Niccoli G, Morrone D, De Luca G, Miraldi F, et al. In-stent restenosis after percutaneous coronary intervention: emerging knowledge on biological pathways. Eur Heart J Open. 2023;3(5):oead083. doi: 10.1093/ehjopen/oead083 37808526 PMC10558044

[5] Liu B, Li M, Liu J, Xie L, Li J, Liu Y, et al. Risk factors and incidence for in-stent restenosis with drug-eluting stent: a systematic review and meta-analysis. Rev Cardiovasc Med. 2024;25(12):458. doi: 10.31083/j.rcm2512458 39742219 PMC11683691

[6] Alfonso F, Coughlan JJ, Giacoppo D, Kastrati A, Byrne RA. Management of in-stent restenosis. EuroIntervention. 2022;18(2):e103–23. doi: 10.4244/EIJ-D-21-01034 35656726 PMC9904384

[7] Guo X, Shen R, Yan S, Su Y, Ma L. Triglyceride-glucose index for predicting repeat revascularization and in-stent restenosis in patients with chronic coronary syndrome undergoing percutaneous coronary intervention. Cardiovasc Diabetol. 2023;22(1):43. doi: 10.1186/s12933-023-01779-7 36864455 PMC9983161

[8] Liu X, Zhang Y, Luo D, Chen B, Lai C, He C, et al. Association between non-high-density lipoprotein cholesterol to high-density lipoprotein cholesterol ratio and cardiometabolic multimorbidity among middle-aged and older adults in China. BMC Public Health. 2025;25(1):570. doi: 10.1186/s12889-025-21757-w 39934789 PMC11817840

[9] Wang B, Li L, Tang Y, Lin T, Wu J, Wang G, et al. Changes in non-high-density lipoprotein to high-density lipoprotein ratio (NHHR) and cardiovascular disease: insights from CHARLS. Lipids Health Dis. 2025;24(1):112. doi: 10.1186/s12944-025-02536-3 40133921 PMC11934592

[10] Cutlip DE, Windecker S, Mehran R, Boam A, Cohen DJ, van Es G-A, et al. Clinical end points in coronary stent trials: a case for standardized definitions. Circulation. 2007;115(17):2344–51. doi: 10.1161/CIRCULATIONAHA.106.685313 17470709

[11] Räber L, Mintz GS, Koskinas KC, Johnson TW, Holm NR, Onuma Y, et al. Clinical use of intracoronary imaging. Part 1: guidance and optimization of coronary interventions. An expert consensus document of the European Association of Percutaneous Cardiovascular Interventions. Eur Heart J. 2018;39(35):3281–300. doi: 10.1093/eurheartj/ehy285 29790954

[12] Mehran R, Dangas G, Abizaid AS, Mintz GS, Lansky AJ, Satler LF, et al. Angiographic patterns of in-stent restenosis: classification and implications for long-term outcome. Circulation. 1999;100(18):1872–8. doi: 10.1161/01.cir.100.18.1872 10545431

[13] Visseren FLJ, Mach F, Smulders YM, Carballo D, Koskinas KC, Bäck M, et al. 2021 ESC Guidelines on cardiovascular disease prevention in clinical practice. Eur Heart J. 2021;42(34):3227–337. doi: 10.1093/eurheartj/ehab484 34458905

[14] Castañer O, Pintó X, Subirana I, Amor AJ, Ros E, Hernáez Á, et al. Remnant cholesterol, not LDL cholesterol, is associated with incident cardiovascular disease. J Am Coll Cardiol. 2020;76(23):2712–24. doi: 10.1016/j.jacc.2020.10.008 33272365

[15] Sehayek D, Cole J, Björnson E, Wilkins JT, Mortensen MB, Dufresne L, et al. ApoB, LDL-C, and non-HDL-C as markers of cardiovascular risk. J Clin Lipidol. 2025;19(4):844–59. doi: 10.1016/j.jacl.2025.05.024 40681368

[16] Li Z, Xu H. Association between non-high-density lipoprotein cholesterol to high-density lipoprotein cholesterol ratio and cardiovascular disease mortality in patients with type 2 diabetes mellitus and diabetic kidney disease. Front Endocrinol (Lausanne). 2025;16:1509752. doi: 10.3389/fendo.2025.1509752 40093754 PMC11906292

[17] Suh SH, Oh TR, Choi HS, Kim CS, Bae EH, Ma SK, et al. Non-high-density lipoprotein cholesterol and progression of chronic kidney disease: results from the KNOW-CKD study. Nutrients. 2022;14(21):4704. doi: 10.3390/nu14214704 36364966 PMC9656579

[18] Duan Y, Yang K, Zhang T, Guo X, Yin Q, Liu H. Association between non-highdensity lipoprotein cholesterol to high-density lipoprotein cholesterol ratio and cardiovascular-kidney-metabolic syndrome: evidence from NHANES 2001-2018. Front Nutr. 2025;12:1548851. doi: 10.3389/fnut.2025.1548851 40093879 PMC11906338

[19] Tan MY, Weng L, Yang ZH, Zhu SX, Wu S, Su JH. The association between non-high-density lipoprotein cholesterol to high-density lipoprotein cholesterol ratio with type 2 diabetes mellitus: recent findings from NHANES 2007-2018. Lipids Health Dis. 2024;23(1):151. doi: 10.1186/s12944-024-02143-8 38773578 PMC11106869

[20] Wu J, Guo J. Non-high-density lipoprotein cholesterol to high-density lipoprotein cholesterol ratio (NHHR) and hypertension in American adults: a NHANES cross-sectional study. Front Physiol. 2024;15:1398793. doi: 10.3389/fphys.2024.1398793 39193442 PMC11348435

[21] Chen L, Wu S, Zheng Y, Shen Y, Sun X, Wang L. The relationship between Non-HDL/HDL ratio and in-stent restenosis in patients with coronary artery atherosclerosis after percutaneous coronary intervention. BMC Cardiovasc Disord. 2025;26(1):19. doi: 10.1186/s12872-025-05400-5 41350986 PMC12798007

[22] Yang Z, Li Y, Yang M, Xu Y, Yao J, Wang K, et al. Exploring the relationship between NHHR and the degree of coronary artery stenosis in patients with acute coronary syndromes. BMC Cardiovasc Disord. 2025;25(1):589. doi: 10.1186/s12872-025-05066-z 40775270 PMC12330120

[23] Li Y, Chen X, Li S, Ma Y, Li J, Lin M, et al. Non-high-density lipoprotein cholesterol/high-density lipoprotein cholesterol ratio serve as a predictor for coronary collateral circulation in chronic total occlusive patients. BMC Cardiovasc Disord. 2021;21(1):311. doi: 10.1186/s12872-021-02129-9 34162320 PMC8223315

[24] Liu M, Pei J, Zeng C, Xin Y, Zhang Y, Tang P, et al. Association of non-high-density lipoprotein cholesterol/high-density lipoprotein cholesterol ratio with cardiovascular outcomes in patients with type 2 diabetes mellitus: Evidence from the ACCORD cohort. Diabetes Obes Metab. 2025;27(1):300–11. doi: 10.1111/dom.16018 39415313 PMC11618286

[25] Zhang L, Fan D, Zhu T, Geng L, Gan L, Ou S, et al. The ratio of non-high-density lipoprotein cholesterol to high-density lipoprotein cholesterol is associated with diabetic kidney disease: a cross-sectional study. PLoS One. 2024;19(11):e0311620. doi: 10.1371/journal.pone.0311620 39602386 PMC11602080

[26] Poznyak AV, Sukhorukov VN, Eremin II, Nadelyaeva II, Gutyrchik NA, Orekhov AN. HDL-based therapy: vascular protection at all stages. Biomedicines. 2023;11(3):711. doi: 10.3390/biomedicines11030711 36979690 PMC10045384

[27] Raza H, Noor T, Umer S, Fatima M, Imran A, Malik N. Relationship between high-density lipoprotein-cholesterol and red cell distribution width in patients with coronary artery disease. Cureus. 2022;14(3):e23132. doi: 10.7759/cureus.23132 35425675 PMC9005557

[28] Shoji T, Masakane I, Watanabe Y, Iseki K, Tsubakihara Y, Committee of Renal Data Registry, Japanese Society for Dialysis Therapy. Elevated non-high-density lipoprotein cholesterol (non-HDL-C) predicts atherosclerotic cardiovascular events in hemodialysis patients. Clin J Am Soc Nephrol. 2011;6(5):1112–20. doi: 10.2215/CJN.09961110 21511840 PMC3087778

[29] Pelliccia F, Zimarino M, Niccoli G. In-stent restenosis after percutaneous coronary intervention: emerging knowledge on biological pathways. Eur Heart J Open. 2023;3(5):oead083. doi: 10.1093/ehjopen/oead083 37808526 PMC10558044

[30] Xu B-Y, Xiang M-X, Wang J. Endothelial progenitor cells and in-stent restenosis. Curr Stem Cell Res Ther. 2015;10(4):364–71. doi: 10.2174/1574888x10666150204150430 25654305

[31] O’Sullivan JF, Martin K, Caplice NM. Microribonucleic acids for prevention of plaque rupture and in-stent restenosis: “a finger in the dam”. J Am Coll Cardiol. 2011;57(4):383–9. doi: 10.1016/j.jacc.2010.09.029 21251577

[32] Zhu P u, Liu H ui, Liu N a. Association between non-HDL to HDL cholesterol ratio and unstable coronary plaque formation in elderly patients with coronary heart disease. Henan Medical Research. 2025;34(01):92–5. doi: 10.3969/j.issn.1004-437X.2025.01.021

[33] Mardi P, Abdi F, Ehsani A, Seif E, Djalalinia S, Heshmati J, et al. Is non-high-density lipoprotein associated with metabolic syndrome? A systematic review and meta-analysis. Front Endocrinol (Lausanne). 2022;13:957136. doi: 10.3389/fendo.2022.957136 36176470 PMC9514792

[34] Tan M-Y, Zhang P, Wu S, Zhu S-X, Gao M. Association between non-high-density lipoprotein cholesterol to high-density lipoprotein cholesterol ratio and serum thyroid function measures: recent Findings from NHANES 2007-2012 and Mendelian randomization. Front Endocrinol (Lausanne). 2025;16:1467254. doi: 10.3389/fendo.2025.1467254 39926348 PMC11802436

[35] Zhang N, Hu X, Zhang Q, Bai P, Cai M, Zeng TS, et al. Non-high-density lipoprotein cholesterol: High-density lipoprotein cholesterol ratio is an independent risk factor for diabetes mellitus: results from a population-based cohort study. J Diabetes. 2018;10(9):708–14. doi: 10.1111/1753-0407.12650 29437292

